# Reported history of childhood trauma, mentalizing deficits, and hypersomnia in adulthood: A mediational analysis in a nonclinical sample

**DOI:** 10.1002/brb3.3363

**Published:** 2024-01-02

**Authors:** Bessat Kalantar‐Hormozi, Shahram Mohammadkhani

**Affiliations:** ^1^ Department of Clinical Psychology, Kharazmi University Faculty of Psychology and Education Tehran Iran

**Keywords:** childhood trauma, emotional abuse, hypersomnia, mentalizing, sleep, structural equation modeling

## Abstract

**Background and objective:**

Existing research has confirmed the link between childhood trauma and poor sleep quality in adulthood. This study focused on the relationship between childhood trauma and hypersomnia specifically, which is understudied. Additionally, childhood maltreatment has been related to mentalizing deficits. The current study examined the role of mentalizing deficits as mediators between childhood trauma and hypersomnia.

**Method:**

The study sample of this cross‐sectional study consisted of 496 individuals, who participated in the online survey, which contained the following measures: Persian version of the Mini Sleep Questionnaire, Reflective Functioning Questionnaire (RFQ‐8), and Childhood Trauma Questionnaire (CTQ).

**Results:**

The results from structural equation modeling indicated that emotional abuse positively predicts hypersomnia. Mediation analysis confirmed that hypo‐mentalizing partially mediates the association between emotional abuse and hypersomnia.

**Conclusion:**

The present study provides primary evidence that experiencing emotional abuse during childhood is associated with hypersomnia in adulthood. This association underlines the importance of prevention. The result from mediation analysis suggests addressing mentalizing impairments in patients with hypersomnia and a history of emotional abuse may be helpful.

## Graphical abstract

Hypo‐mentalizing relates history of Emotional Abuse to hypersomnia.

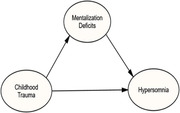

Sleep is essential for optimal health, cognition, immune system well‐being, memory consolidation, endocrine activity, and cardiovascular health (Stuck et al., 2021). Available research has made it clear that poor sleep quality is comorbid with many psychological disorders and impacts treatment outcomes negatively (Horenstein et al., 2019; Zalta et al., 2013). Thus, addressing sleep disturbances can be an important target for psychotherapeutic interventions.

The link between early life stress (e.g., childhood trauma) and sleep problems in adulthood is very well documented (Brown et al., 2022; Kajeepeta et al., 2015; Lewin et al., 2019; Lo Martire et al., 2020). Identifying potential mechanisms by which childhood trauma provokes sleep disturbances will boost trauma‐informed psychological treatments. Previous findings suggest protentional mediators in the aforementioned association such as perceived stress (Cardoso et al., 2018), neuroticism (Ramsawh et al., 2011), depression and anxiety (Park et al., 2021), and psychological distress (McPhie et al., 2014). Mentalizing, one of the psychological capacities, which is impacted by childhood maltreatment has not been studied well in relation to sleep disturbances. Mentalizing is the imaginative activity people use to perceive and interpret behavior based on intentional mental states (Sharp & Bevington, 2022). Luyten et al. (2020), recommend that mentalizing, as a transtheoretical and transdiagnostic concept, can explain vulnerability to psychopathology and its treatment. Two main mentalizing deficits that are currently captured by available measures are hyper‐mentalizing and hypo‐mentalizing. Hyper‐mentalizing refers to interpreting thoughts and behavior far beyond the pertinent context. Hypo‐mentalizing is the predisposition to develop poor or simplistic explanations for behavior or intentions (Sharp & Bevington, 2022). Previous findings confirm the mediational role of mentalizing in the association between childhood maltreatment and psychological disorders (Li et al., 2020; Weijers et al., 2018).

Furthermore, most of the previous studies that connect childhood trauma to sleep problems have either addressed sleep issues without differentiating between different sleep disorders or have focused on insomnia, perhaps because insomnia is more prevalent (Stuck et al., 2021) or, because of expectations that alertness and hyperarousal caused by the body's trauma response is more probable to be accompanied by symptoms of insomnia (Bader et al., 2007; Riemann et al., 2010). Hypersomnia is defined as increased daytime sleepiness despite sufficient or even very long night sleep (Stuck et al., 2021) and is associated with health‐related, socioeconomic consequences for patients, their partners, and society (Jennum et al., 2014). Okada et al. (2018) studied sleep disturbances using data from 273 abused children and adolescents (age range: 4 to 15 years) and reported daytime sleepiness in 10% of the study sample. Chapman et al. (2011) used data from the adverse childhood experiences (ACE) study, a retrospective cohort study of 17,337 adults in California and concluded that compared to persons with an ACE score of 0, those with an ACE score ⩾5, are 2.0 (95% CI: 1.7−2.3) times more likely to report feeling tired even after a good night's sleep.  In a study by April‐Sanders et al. (2021), the association between childhood adversities and indicators of sleep disturbance was examined in a longitudinal cohort of Puerto Rican youth. The results demonstrated that in 10−16 year‐olds, having ≥ 4 childhood adversities compared with 0 adversities was related to higher prevalence of daytime sleepiness. Also, daytime sleepiness occurred in 5% to 6% of 5‐ to 9‐year‐olds and 8%−12% in older children. On the contrary, Luo et al. (2022) studied the correlation between childhood trauma and hypersomnia in patients with depression and did not find it significant (*r* = .081, *p* = .175). Although studies mentioned above have explored the relationship between childhood trauma and hypersomnia, this relationship is still understudied and therefore was the focus of the present study.

This study aims to further our understanding of the relationship between childhood trauma, mentalizing deficits, and hypersomnia using structural equation modeling. For this purpose, the following hypotheses were explored: (a) childhood trauma positively predicts hypersomnia in adulthood, (b) childhood trauma history and mentalizing deficits are positively correlated, (c) mentalizing deficits positively predict hypersomnia, and (d) the association between childhood trauma history and hypersomnia is mediated by mentalizing deficits. Figure 1 shows the conceptualized model of this study.

**FIGURE 1:**
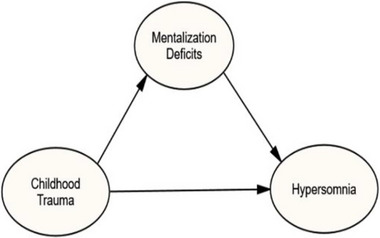
The conceptualized model.

## 1 METHODS

### 1.1 Participants and procedure

The study design was a cross‐sectional questionnaire‐based correlational study. Structural equation modeling was used to explore the proposed hypotheses. Data collection was executed via an online survey shared on multiple social media platforms (Instagram stories, Twitter, and talk forums with Iranian audience) from November 22, 2022 to February 19, 2023. Research goals and inclusion/exclusion criteria were explained on the first page of the survey. Confidentiality was guaranteed through anonymous sampling and participants were asked to provide consent to participate before taking the survey. All fields were required to be completed before data submission was complete therefore missing data was not an issue during the final analysis. Inclusion criteria included (1) being an adult (it was specified 22 years old and older) and (2) being willing to complete the research questionnaires voluntarily. Exclusion criteria included (1) being under age 22 years and (2) taking medications that cause sleepiness.

### 1.2 Measures

#### 1.2.1 Demographic questionnaire

The participants completed information about their educational level, age, marital and employment status.

#### 1.2.2 Childhood trauma

The Childhood Trauma Questionnaire (CTQ) is a brief, 28‐item self‐report questionnaire used for screening for childhood maltreatment in adults retrospectively (Bernstein et al., 2003).

CTQ comprises five subscales: Emotional Abuse, Physical Abuse, Sexual Abuse, Emotional Neglect, and Physical Neglect. Items are scored from “never true” (score 1) to “very often true” (score 5). Seven items are reverse scored (2, 5, 7, 13, 19, 26, and 28). The validity and reliability of the Persian version of CTQ were evaluated by Garrusi and Nakhaee (2009) who reported acceptable psychometric properties. In this study, Cronbach's α for the subscales was as follows: Emotional Abuse = .81, Sexual Abuse = .82, Emotional Neglect = .87, Physical Neglect = .54, and Physical Abuse = .56.

#### 1.2.3 Mentalizing

Mentalizing was assessed using the Reflective Functioning Questionnaire (RFQ), which is a self‐report measure developed by Fonagy et al. (2016) for measuring mentalizing. The brief version of RFQ (RFQ‐8) comprises 8 items such as “People's thoughts are a mystery to me” that are rated on a 7‐point Likert scale ranging from “strongly disagree” to “strongly agree” and two subscales (RFQ‐U or hypo‐mentalizing and RFQ‐C or hyper‐mentalizing) are produced after scoring. Six of eight items are used jointly in both subscales but with different scoring. In previous research (Fonagy et al., 2016), the RFQ‐U and RFQ‐C subscales have shown acceptable internal consistency in a nonclinical sample (Cronbach's α = .63 and .67, respectively). In the Persian version of this measure (Seyed Mousavi et al., 2021), internal consistency was satisfactory for both RFQ‐C (Cronbach's α = .7) and RFQ‐U (Cronbach's α = .62). The test–retest correlation coefficient was .78 for RFQ‐U and .81 for RFQ‐C. In the current study, Cronbach's α value for the hyper‐mentalizing and hypo‐mentalizing subscales was respectively .81 and .75.

#### 1.2.4 Hypersomnia

The current version of Mini Sleep Questionnaire (MSQ), which measures both hypersomnia and insomnia, was originally developed by Zoomer et al. (1985). MSQ contains 10 items in total (4 related to insomnia and 6 related to hypersomnia), each using a 7‐point Likert scale (1, never; 7, always). Sample items include “Do you feel tired after waking up in the morning?” and “Do you wake up during the night?” Flavinga et al. (2011) reported Cronbach's α value of .77 for MSQ. The reliability and validity of the Persian version of MSQ (MSQ‐P) were studied by Hosseini et al. (2020) who reported a Cronbach α value of .75 and a test–retest correlation coefficient of .91 for this scale. The hypersomnia subscale of this measure (items 4, 5, 6, 8, 9, 10 of the MSQ‐P) was used in the current research. The internal consistency (Cronbach's α value) for the hypersomnia subscale was .6.

### 1.3 Statistical analysis

This study benefits from Structural equation modeling (SEM) for data analysis. SEM is a multivariate quantitative technique, which combines path models and confirmatory factor models to depict relationships among variables, and test theoretical models hypothesized by the researcher (Kline, 2015; Thakkar, 2020). The goal of SEM analysis is to determine the extent to which the theoretical model is supported by sample data. SEM begins with model specification, which involves the development of a conceptual model, which defines the variables and their relationships based on available literature and theories (see Figure 1). The next step is model Identification. For a model to be identified, the model should have nonnegative degrees of freedom (Davvetas et al., 2020). The third step involves data collection and addressing the issues of sample size, outliers, normality, and missing data. The next steps are conducting confirmatory factor analysis for each variable and testing the measurement and structural models for insignificant paths and checking model fit by examining the model fit indices (as reported in the Section 2) (Weston & Gore et al., 2006). The final step of structural equation modeling is model modification where the researcher refines the model if needed (Thakkar, 2020).

Mediation analysis attempts to explore whether the relationship between an independent variable (exogenous variable) and an outcome (endogenous variable) is fully or partially explained by a mediator variable. According to Baron and Kenny (1986), if an independent variable predicts the outcome variable significantly, and a mediator variable (which is related significantly to both the predictor and outcome variables) is inserted in the analysis what happens to the relationship between the predictor and the outcome clarifies the role of the mediator variable: if the relationship is no longer significant then we are dealing with a full mediation model; if the relationship is still significant but the regression weight is lower than before then the relationship is only partially affected by the mediator (Baron & Kenny, 1986).

Descriptive data analysis was performed in SPSS 26 for Windows. Hypothesis testing and mediation analysis were conducted in AMOS 24 with structural equation modeling. All five childhood trauma subscales were entered into the model as exogenous variables, hypersomnia was placed as the endogenous variable, and hypo‐mentalizing and hyper‐mentalizing were included as mediators. Consequently, based on the results acquired from the structural model, a mediation analysis was performed with emotional abuse as the exogenous variable and hypo‐mentalizing as the mediator.

## 2 RESULTS

### 2.1 Sample characteristics

A total of 496 participants (424 females and 72 males) were entered into the analysis. The minimum age in the sample was 22 and the maximum age was 63 (MD = 30.03 SD = 6.36). 62.3% (*n* = 309) were married, 37.3% (*n* = 185) were single and 0.4% (*n* = 2) were widowed. 35.5% were employed, 47.4 unemployed%, 16.3% were students, and 0.8% reported to be retired. 23.2% (*n* = 115) were high school graduates, 47.2% (*n* = 234) had a bachelor's degree, 19.2% (*n* = 95) held a master's degree, 5.4% (*n* = 27) held PhD degrees, and 5.0% (*n* = 25) reported their education level to be less than a high school diploma.

### 2.2 Descriptive statistics

Table 1 reports the correlations between the study variables as well as means and standard deviations. Collected data were normally distributed (skewness values were in the range of −2 to +2 and kurtosis values were between −7 to +7). The values for skewness and kurtosis are available in Appendix A. Mahalonobis distance measure (*D* ^2^) was used to detect multivariate outliers. *D* ^2^/*df* was 3.05 (less than 4, so no multivariate outliers were detected; Hair et al., 2010). Table 1 presents the descriptive characteristics of the variables.

**TABLE 1:** Means, standard deviations, and Pearson's correlations between study variables.

Variable	Mean	Std. deviation	1	2	3	4	5	6	7	8
1 Emotional.abuse	10.39	4.85	–							
2 Hypo‐mentalizing	24.23	7.44	.27^**^	–						
3 Hyper‐mentalizing	23.29	8.72	.27^**^	.91^**^	–					
4 Hypersomnia	18.87	5.99	.30^**^	.32^**^	.33^**^	–				
5 Physical abuse	7.12	3.65	.66^**^	.15^**^	.15^**^	.21^**^	–			
6 Sexual abuse	7.56	4.35	.36^**^	.05	.05	.10^**^	.35^**^	–		
7 Emotional neglect	11.66	5.01	.70^**^	.24^**^	.22^**^	.22^**^	.46^**^	.27^**^	–	
8 Physical neglect	8.05	3.25	.54^**^	.22^**^	.23^**^	.27^**^	.42^**^	.26^**^	.59^**^	–

** Correlation is significant at the .01 level (one‐tailed).

### 2.3 Measurement model

The measurement model and the structural model were analyzed via structural equation modeling in AMOS. Confirmatory factor analysis was performed for each of the latent variables before the assessment of the measurement model. Items 4 (msq4) and 6 (msq6) were dropped from the hypersomnia scale due to low factor loadings (less than .5). Item 7 of the RFQ questionnaire, which was an indicator for the hypo‐mentalizing subscale, was negative and was also dropped. For achieving a better model fit, covariances were added between the indicators. The fit indices for the measurement model were as follows: RMSEA = .05, CMIN/DF = 2/66 (*p* = .00), CFI = .9, and IFI = .9.

### 2.4 Structural model

The fit indices showed the structural model provided a good fit with RMSEA = .05, CMIN/DF = 2/66 (*p* = .00), CFI and IFI both = .9 and TLI, GFI close to .9 (respectively .87 and .84). The predictors explained 26% of the variance in hypersomnia. The significance of regression weights was checked to evaluate the hypotheses of the research. The first hypothesis stated that a history of childhood trauma is associated with hypersomnia. Emotional abuse was the only category of childhood trauma that significantly predicted hypersomnia (β = .41, *p* = .03). The paths from physical abuse (β = –.16, *p* = .18), emotional neglect (β = –.16, *p* = .34), sexual abuse (β = .01, *p* = .08) and physical neglect (β = .2, *p* = .15) to hypersomnia were not statistically significant.

The second hypothesis proposed that a history of childhood trauma correlates with mentalizing deficits. Results show except for the covariation between sexual abuse and hypo‐mentalizing, which was not significant (β = .07, *p* = .15) all other categories of childhood trauma had a positive and significant covariation with hypo‐mentalizing. The covariation of hyper‐mentalizing with emotional neglect, emotional abuse, physical abuse, sexual abuse, and physical neglect was negative but significant.

Investigating the third hypothesis demonstrated that hyper‐mentalizing did not predict hypersomnia (β = –.07, *p* = .38). However, hypo‐mentalizing predicted hypersomnia positively and significantly (β = .21, *p* = .02).

**FIGURE 2:**
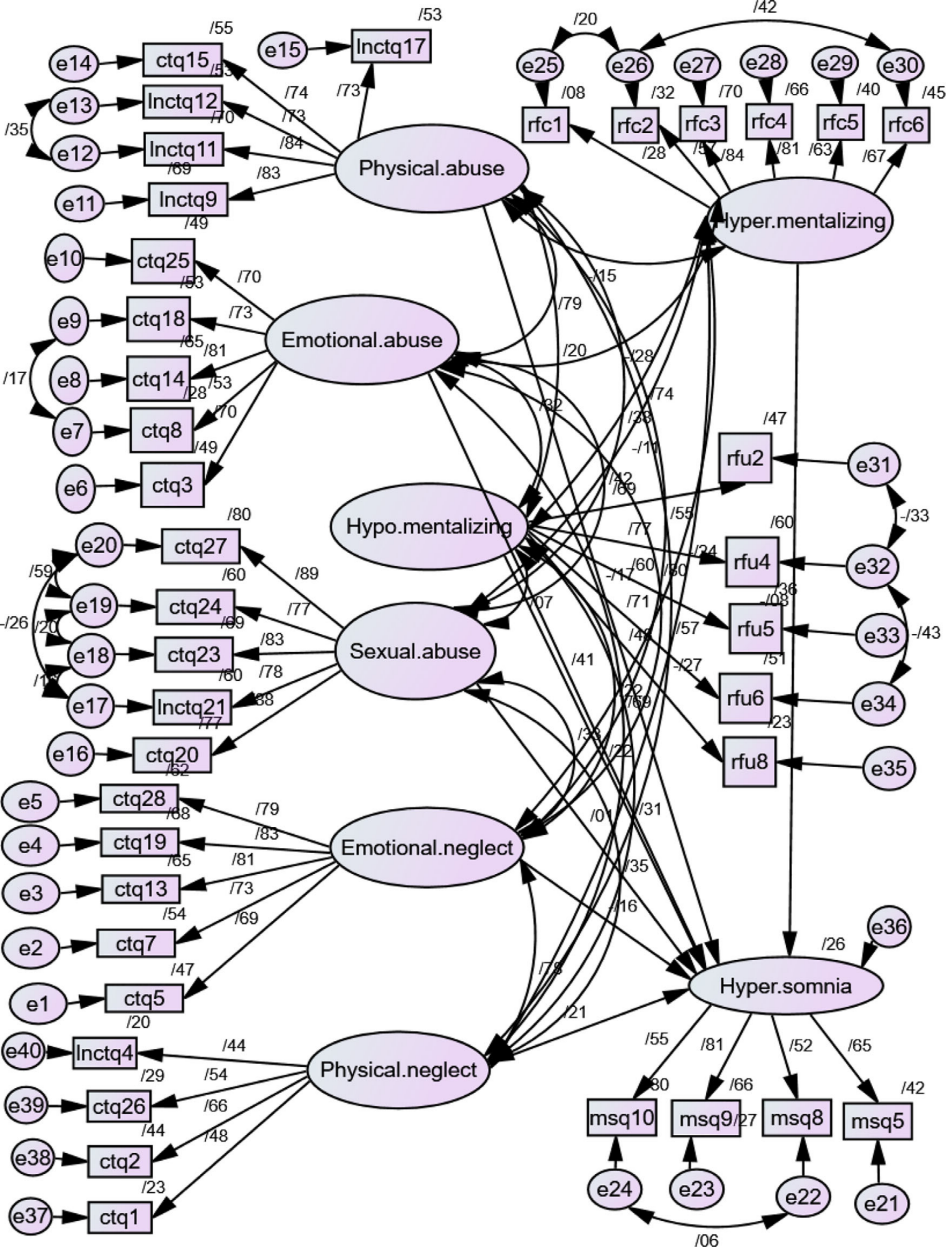
The structural model in the standardized mode.

**FIGURE 3:**
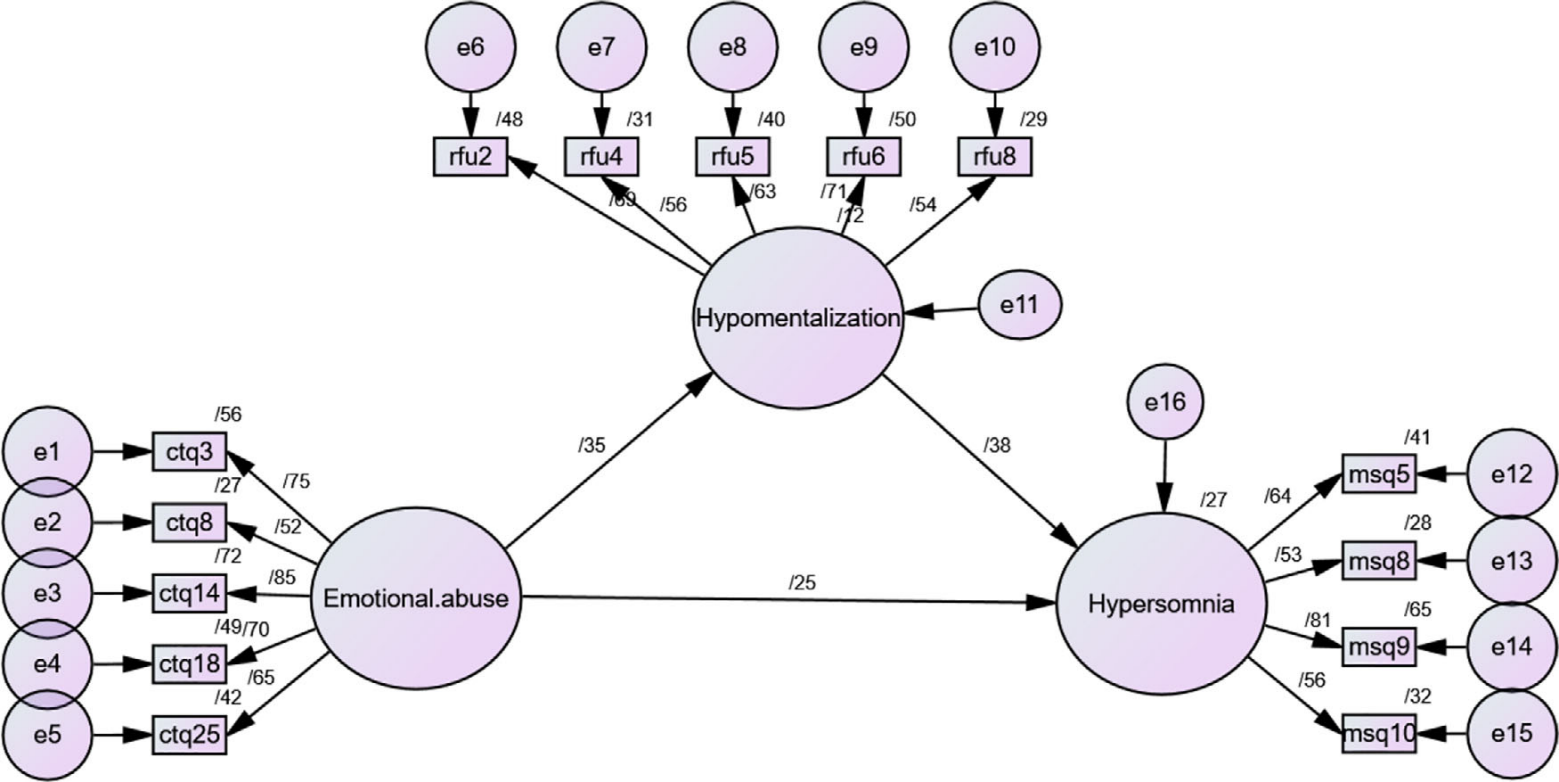
The full mediation model in standard mode.

The fourth hypothesis suggested a mediational role for mentalizing deficits in the association between a history of childhood trauma and hypersomnia. According to the acquired results reported above, a mediation model was investigated based on Baron and Kenny's method in which hypo‐mentalizing was set as the mediator variable and emotional abuse and hypersomnia were the exogenous and endogenous variables respectively. Figure 2 presents the structural model in the standardized mode. The full mediation model is shown in Figure 3.

Higher fit indices and lower AIC (Akaike Information Criterion) propose that the full mediation model has a better fit than the direct and indirect models. However, the path from emotional abuse to hypersomnia was significant in the direct model (β = .37, *p* = .001) and the full mediation model (β = .24, *p* = .001) suggesting a partial mediational effect for hypo‐mentalizing. Table 2 summarizes model fit indices.

**TABLE 2:** Model fit summary for the direct, indirect, and full mediation models.

Fit index	Direct model	Indirect model	Full mediation model
CMIN/DF	3/52	2/68	2/46
DF	76	75	74
*p*	.00	.00	.00
AGFI	.90	.92	.92
GFI	.92	.94	.94
CFI	.90	.93	.94
IFI	.90	.93	.94
PNFI	.72	.74	.74
RMSEA	.07	.05	.05
AIC	325/75	261/62	244/08

CMIN/DF, normed chi‐square; DF, degree of freedom; *p, p*‐value; AGFI, Adjusted Goodness of Fit Index; GFI, Goodness of Fit Index; CFI, Comparative Fit Index; IFI, Incremental Fit Index; PNFI, Parsimonious Normed Fit Index; RMSEA, root mean square error of approximation; AIC, Akaike Information Criterion.

## 3 DISCUSSION

This research had two ultimate goals: first, to examine the association between different categories of childhood trauma and hypersomnia, and second, to check whether mentalizing deficits have a mediational role in this association. Overall, the results indicated that emotional abuse was the only form of childhood trauma that predicted hypersomnia. The proposed mediational model demonstrated a good fit and hypo‐mentalizing, a form of mentalizing deficit, partially mediated the relationship between emotional abuse and hypersomnia.

This study documented a significant, positive link between emotional abuse and hypersomnia. This finding accords with prior research, which also establishes childhood trauma as a predictor of sleep issues. Cardoso et al. (2018) also found emotional abuse to be the only category of childhood trauma that was a significant predictor of sleep disturbances. A possible explanation is that childhood emotional abuse highly predicts depressive symptoms, and, hypersomnia can be a byproduct of depression (Christ et al., 2019; Dauvilliers et al., 2013). Another explanation may be that distinct childhood trauma categories may foster different coping styles. Whatts et al. (2020) studied avoidant coping in separate childhood adversities and found only emotional abuse uniquely explained experiential avoidance. Previous research suggests that avoidant coping styles would be more likely to lead to excessive sleepiness (Sadeh & Gruber, 2002).

In this study, physical abuse, physical neglect, sexual abuse, and emotional neglect did not predict hypersomnia, which is in agreement with studies by Laskemoen et al. (2021) and also Luo et al. (2022). The outcomes of this study imply that distinct trauma subtypes may interact differently with sleep mechanisms as physiological responses to stress. Prior research is already in favor of this finding. Miller et al. (2007) discuss separate types of traumas that may demand different physiological responses to facilitate adaptation. Kuhlman et al. (2015) state different types of childhood trauma exposure are related to diverse irregularities in hypothalamic‐pituitary‐adrenal axis (HPA) functioning, which modulates many physiological aspects, such as the wake‐sleep cycle. Kuhlman et al. (2015) also suggest that trauma subtypes that can be categorized as “unpredictable” such as community violence may have a different impact on the stress response as opposed to “predicted” trauma such as repeated physical abuse, which tends to accumulate over time. In this regard, recent neuroscientific research, which discusses the structural and functional brain abnormalities associated with exposure to different childhood trauma subtypes and sleep also justify our results (Fuligni et al., 2021; Yu et al., 2022). These findings may provide insight for screening and interventions. Professionals can help childhood emotional abuse survivors understand how emotional abuse, has likely contributed to their hypersomnia.

Additionally, results indicated all categories of childhood trauma except sexual abuse had significant positive covariations with hypo‐mentalizing. This finding is in agreement with the mentalization theory, which posits childhood maltreatment undermines the development of mentalizing. Similar results were obtained in previous research, which also found positive associations between hypo‐mentalizing and history of trauma (Doba et al., 2022; Huang et al., 2020; Wagner‐Skacel et al., 2022). Contrary to our results, Ensink et al. (2015) found sexual abuse was significantly associated with mentalizing difficulties. This inconsistency may be due to different sample characteristics, for example age, reluctance to report sexual abuse due to cultural issues, or even lower rate of sexual abuse occurrence in the study sample.

Furthermore, the findings revealed hypo‐mentalizing positively predicted hypersomnia. Although this is a new finding in terms of investigating the mentalization construct, prior research has acknowledged and confirmed the association between disability in interpreting states of mind in oneself and others and sleep problems (Alimoradi et al., 2022). Bauermann et al. (2008) studied excessive sleepiness in young adults with alexithymia (a personality trait that overlaps with affective hypo‐mentalizing) and found alexithymic adults scored significantly higher in excessive sleepiness than their nonalexithymic counterparts. In the present study, hyper‐mentalizing, which entails excessive interpretation of behaviors and intentional states of mind did not predict hypersomnia, which was very much an expected result because “excessive interpretation” might involve rumination and rumination best predicts insomnia rather than hypersomnia (Frøjd et al., 2022; Galbiati et al., 2018).

Lastly, this research suggested that the link between emotional abuse and hypersomnia is partially mediated by hypo‐mentalizing. This result is in harmony with earlier research findings, which suggest deficits in mentalizing mediate the relationship between childhood maltreatment and psychological/somatic symptoms (Li et al., 2020; Redono & Luyten, 2018; Riem et al., 2018 Schwarzer et al., 2021). The results show a partial mediation, suggesting both direct and indirect paths are significant and although hypo‐mentalizing as a psychological construct does not solely account for the link between emotional abuse and hypersomnia, the results support its mediating role. The approved mediating role of hypo‐mentalizing in the current study is noteworthy clinically. Exploring bodily symptoms and sleep disruptions together with the patient is an important part of most trauma therapies. Trauma‐Focused Acceptance and Commitment Therapy (TFACT) (Harris, 2021), Mindfulness based Stress reduction (MBSR) (Kabat‐Zinn & Hanh, 2009), somatic therapies such as Somatic Experiencing (Levine, 2010), and Sensorimotor psychotherapy (Ogden et al., 2006) all attend to body sensations and sleep issues.

By confirming the mediating role of mentalizing deficits in the relationship between childhood emotional abuse and hypersomnia in adulthood, the results of this study allow researchers to put a step forward in applying mentalization theory to trauma interventions when sleep issues are present.

### 3.1 Strengths, limitations, and implications for further research

This study offers encouraging primary evidence that targeting mentalizing deficits may benefit patients with a history of childhood trauma and poor sleep. It is to be noted that studies that investigate mentalizing and sleep, or, study hypersomnia as a consequence of childhood trauma are very rare. Therefore, the current research adds to the available findings in this field. Another strength of the current study was the sample size (*n* = 496) which was satisfactorily large considering the number of survey items.

It is also necessary to point out quite a few limitations of the present study and important areas for future research. First of all, although longitudinal research designs are more favorable when it comes to mediation analysis, a cross‐sectional design was used, which limits causal inference. Second, self‐report measures were used to collect data retrospectively, which (similar to all other self‐report measures) have the potential to cause response bias and were also subject to recall bias. Meanwhile, the present study did not control for gender or separate clinical/nonclinical population, which may remain an important area for future research. Since the sample is limited to Iranians only and lacks diversity, any generalization should be carried out with caution. As for the study measures, while the Childhood Trauma Questionnaire is a validated scale, which is a strength, it does not include other potential traumas, such as exposure to violence in the home or neighborhood, death of a loved one, or separation from a loved one.

Nevertheless, the results can expand our knowledge of the potential mechanisms by which childhood trauma is related to sleep disturbances and have implications for the treatment of patients who suffer from hypersomnia.

## 4 CONCLUSION

This research that investigated the relationship between childhood trauma, hypersomnia, and mentalizing found a link between experiencing emotional abuse during childhood and hypersomnia in adulthood. Results also indicate that hypo‐mentalizing partially mediates this link; therefore, this study further supports mentalizing as a psychological capacity that can help health promotion.

## AUTHOR CONTRIBUTIONS

**Bessat Kalantar‐Hormozi**: conceptualization; data curation; formal analysis; writing—original draft; writing—review and editing. **Shahram Mohammadkhani**: conceptualization; supervision; writing—review and editing.

## CONFLICT OF INTEREST STATEMENT

The authors declare no conflict of interest.

## FUNDING

No public or private organization has provided funding for this research.

## ETHICS STATEMENT

This study was approved by the Department of clinical psychology, Faculty of Psychology and Education Kharazmi university and was exempt from obtaining ethics approval number on the basis that the study was a none‐interventional, none‐experimental survey study and did not involve children or animals. All participants provided written informed consent prior to taking part in the study and agreed to publication of the results in the form of a research article. The participants completed the survey voluntarily and anonymously, and confidentiality of data was assured.

### PEER REVIEW

The peer review history for this article is available at https://publons.com/publon/10.1002/brb3.3363.

## Data Availability

The data analyzed for the results of this research are available from the corresponding author upon reasonable request.

## APPENDIX A The skewness and kurtosis values.

### Assessment of normality

**Table:**

Variable	Min	Max	Skew	Kurtosis
**lnctq4**	/000	/602	2/113	2/956
**ctq26**	1/000	5/000	1/456	/896
**ctq2**	1/000	5/000	1/487	1/354
**ctq1**	1/000	5/000	1/318	/921
**rfu8**	/000	3/000	/567	−1/262
**rfu6**	/000	3/000	1/424	/520
**rfu5**	/000	3/000	/720	–/948
**rfu4**	/000	3/000	/487	−1/470
**rfu2**	/000	3/000	1/371	/418
**rfc6**	/000	3/000	/314	−1/524
**rfc5**	/000	3/000	1/085	–/455
**rfc4**	/000	3/000	1/041	–/493
**rfc3**	/000	3/000	/727	−1/106
**rfc2**	/000	3/000	/500	−1/289
**rfc1**	/000	3/000	1/127	–/074
**msq8**	1/000	7/000	/869	/000
**msq10**	1/000	7/000	/651	–/715
**msq9**	1/000	7/000	–/040	−1/131
**msq5**	1/000	7/000	–/192	–/809
**ctq27**	1/000	5/000	1/880	2/461
**ctq24**	1/000	5/000	2/332	4/374
**ctq23**	1/000	5/000	1/977	3/206
**lnctq21**	/000	/699	2/235	3/821
**ctq20**	1/000	5/000	1/405	1/094
**lnctq17**	/000	/699	2/811	6/553
**ctq15**	1/000	5/000	1/447	1/074
**lnctq12**	/000	/699	1/589	1/128
**lnctq11**	/000	/699	1/642	1/335
**lnctq9**	/000	/699	2/210	3/651
**ctq25**	1/000	5/000	/575	–/994
**ctq18**	1/000	5/000	1/142	–/005
**ctq14**	1/000	5/000	/931	/004
**ctq8**	1/000	5/000	1/183	/261
**ctq3**	1/000	5/000	/931	–/124
**ctq28**	1/000	5/000	/674	–/590
**ctq19**	1/000	5/000	/488	–/642
**ctq13**	1/000	5/000	/731	–/270
**ctq7**	1/000	5/000	/658	–/476
**ctq5**	1/000	5/000	/425	−1/067
**Multivariate**				288/465
